# Feasibility, safety, and adherence of a remote physical and cognitive exercise protocol for older women

**DOI:** 10.1055/s-0044-1785690

**Published:** 2024-04-23

**Authors:** Cristiane Peixoto, Maria Niures Pimentel dos Santos Matioli, Satiko Andrezza Ferreira Takano, Maurício Silva Teixeira, Carlos Eduardo Borges Passos Neto, Sonia Maria Dozzi Brucki

**Affiliations:** 1 Universidade de São Paulo, Faculdade de Medicina, Departamento de Neurologia, São Paulo SP, Brazil.; 2 Hospital Estadual Guilherme Álvaro, Santos SP, Brazil.

**Keywords:** Feasibility Studies, Exercise, Telemonitoring, Aged, Estudos de Viabilidade, Exercício, Telemonitoramento, Idoso

## Abstract

**Background** Population aging and the consequences of social distancing after the COVID-19 pandemic make it relevant to investigate the feasibility of remote interventions and their potential effects on averting functional decline.

**Objective** (1) To investigate the feasibility, safety, and adherence of a remote protocol involving physical and cognitive exercises for older women with normal cognition; (2) to examine its effects on cognitive and well-being variables.

**Methods** Twenty-nine women (age ≥ 60 years old) were randomized into experimental group (EG; *n* = 15) and control group (CG; *n* = 14). The EG performed a 40-minute session of cognitive and physical exercises, and CG performed a 20-minute stretching session. Both groups performed 20 sessions via videoconference and 20 on YouTube twice a week. The Mini-Mental State Examination, Verbal Fluency Test, Digit Span (direct an inverse order), Geriatric Depression Scale (GDS), and Well-being Index (WHO-5) were applied in pre- and post-interventions by phone.

**Results** Overall adherence was 82.25% in EG and 74.29% in CG. The occurrence of adverse events (mild muscle pain) was 33.3% in EG and 21.4% in CG. The EG improved verbal fluency and attention ( *p* ≤ 0.05); both groups had improved depressive symptoms.

**Conclusion** The present study met the pre-established criteria for feasibility, safety, and adherence to the remote exercise protocol among older women. The results suggest that a combined protocol has more significant potential to improve cognitive function. Both interventions were beneficial in improving the subjective perception of well-being.

## Resumo

**Antecedentes** O envelhecimento populacional e as consequências do isolamento social após a pandemia de COVID-19 tornaram relevante investigar a viabilidade, segurança e aderência de intervenções remotas e potenciais efeitos para prevenir declínios funcionais.

**Objetivo** (1) Investigar a viabilidade, segurança e aderência de um protocolo remoto de exercícios físicos e cognitivos; (2) investigar os possíveis efeitos sobre variáveis de cognição e de bem-estar.

**Métodos** Vinte e nove mulheres foram randomicamente divididas em grupo experimental (GE; *n* = 15) e grupo controle (GC; *n* = 14). O GE realizou sessões de 40 minutos de exercícios físicos e cognitivos e o GC, 20 minutos de alongamentos. Totalizaram 20 sessões por videoconferência e 20 pelo YouTube, duas vezes por semana. O Teste de Fluência Verbal, o Teste de Dígitos (ordem direta e inversa), a Escala de Depressão Geriátrica (GDS) e o Índice de Bem-Estar (WHO-5) foram aplicados no pré e pós-intervenção, por telefone e formulário digital.

**Resultados** A aderência geral média foi de 82,25% no GE e 74,29% no GC. A ocorrência de eventos adversos (dores musculares leves) foi de 33,3% no GE e 21,4% no GC. O GE teve melhora em fluência verbal e atenção ( *p* ≤ 0.05) e ambos os grupos tiveram melhora significativa nos sintomas depressivos.

**Conclusão** O presente estudo atendeu aos critérios preestabelecidos para a viabilidade, segurança e aderência do programa oferecido entre idosas. Os resultados sugerem que o protocolo combinado tenha maior potencial de aprimorar funções cognitivas. Ambas as intervenções foram benéficas para a percepção subjetiva de bem-estar.

## INTRODUCTION

Increasing age is considered the most relevant risk factor for neurological diseases, so population aging is a crucial problem in global public health. 1 2 3 The 2020 Lancet Dementia Prevention report pointed out that risk factors for developing neurodegenerative diseases are associated with about 40% of cases of dementia worldwide, which could be avoided or postponed, especially in developing countries. 4 Almost half of the risk factors mentioned in this report can be controlled by the regular and systematic practice of physical activities. 5

Physical activity has been considered an essential modifiable factor in lifestyle. It is associated with increased longevity, functional capacity, improved cognitive functions, and reduced dementia. 6 7 8 9 10 11 Experts in cognitive aging point out the need to stimulate neuroplasticity through the learning process, in addition to the positive effect of physical exercise due to the greater expression of neurotrophins such as the BDNF. 12 The combined interventions of physical exercises with cognitive stimulation performed in sedentary posture or activities requiring the use of technology (such as exergames) can improve cognitive functions. 13 14 15 16 17

The COVID-19 pandemic has had consequences on people's health, impacting anxiety and depressive symptoms. 18 About 80 to 95% of fatal cases from COVID-19 in Europe and Asia were among people over 60 years old. 19 Social distancing reduces physical activity levels, which can negatively affect the physical and mental health of older individuals. 20 21 The feeling of loneliness and the level of physical activity should represent a target of scientific research since they are related to mental health, are influenced by restrictions and isolation policies, and are potentially modifiable by social interventions. 22

Remote physical exercise protocols were well carried out before the COVID-19 pandemic. 14 23 24 One of the main barriers to the systematic practice of physical activities among older individuals is the travel to training centers, in addition to the fact that in-person interventions demand higher costs and are less likely to be administered on a large scale. 25

The conditions for home-based physical and cognitive exercises are different from traditional models. Therefore, the present study aimed to evaluate the feasibility, safety, and adherence of a remote exercise protocol for older women with normal cognition and compare the possible effects of two interventions on cognitive and well-being variables. The motive is justified by the lack of knowledge concerning interventions that do not require tablets or software and allow human interaction in real-time, extending the hypothesis that both interventions are feasible and provide a positive impact on well-being variables, but combined intervention presents a higher advantage on cognitive variables.

## METHODS

### Population and ethical matters

The Ethics and Research Committee of the Medical School of the University of São Paulo approved the present study (n° 4.715.520). Participants have been recruited through a Facebook ad aimed at women over 60 years old. Considering the small sample of previous feasibility studies, 14 15 16 17 23 mainly for statistical analysis purposes, we decided to recruit only women. The G*Power software was used to calculate the sample size, and it was 39 individuals in each group.

Three hundred reais were invested in the ad, bringing 150 people to the initial WhatsApp group. The exclusion criteria were to be unable to connect to the test session on Google Meet, answering “yes” to any of questions 3, 4, and 7 of the Physical Activity Readiness Questionnaire (PAR-Q), 26 that suggest increased cardiovascular risk, self-declaration of the previous diagnosis of dementia or neurological diseases, unstable arterial hypertension, or anxiety disorders. Nine people were excluded by these criteria, and ninety-one left the WhatsApp group after these initial questionnaires.

Therefore, 50 women were divided by stratified randomization using Excel software, by the criterion of level of physical activity practice measured by the International Physical Activity Questionnaire (IPAQ) 27 into an experimental (EG) and control group (CG). The IPAQ measures the amount of time spent in daily physical activity. Based on the criteria established by the World Health Organization (WHO), 1 participants were classified as active, inactive, or insufficiently active.

All volunteers signed a free informed consent form. During the first month of interventions, 21 people dropped out of the study. Twenty-nine women (EG = 15; CG = 14) from 17 cities in 9 Brazilian states participated in the study.

### Study design

After randomization, the initial WhatsApp group was divided into the experimental group (EG) and control group (CG) to access respective interventions, clarify doubts, and allow interaction.

The 10-Cognitive Screen (10-CS) 28 and Functional Activities Questionnaire (FAQ) 29 were used to screen cognitive status. The following tests were used to measure well-being and cognitive variables and performed in pre- and post-interventions. Well-being variables were measured by a Microsoft form containing the Geriatric Depression Scale (GDS), 30 indicating the presence of depressive symptoms; and by the Well-Being Index (WHO-5), 31 a scale in which the individual agrees with positive statements about their health from 0 (worst result) to 5 (best result).

Cognitive variables were evaluated by physicians blinded to randomization through a series of phone-based tests: the Verbal Fluency Test (VFT) 32 to evaluate executive functions and verbal fluency; the forward and backward digit span 33 to assess attention, short-term memory, and verbal working memory; and the 22-point Mini-Mental State Examination (MMSE), validated for telephone use, 34 to evaluate overall cognitive functions.

A self-report form to assess adherence to YouTube sessions and the occurrence of adverse events was used in the middle and at the end of the intervention. The researcher recorded adherence to the live sessions on Google Meet in real time. The adverse events were classified as mild (grade 1), moderate (grade 2), and severe (grades 3 to 5), according to Common Terminology Criteria for Adverse Event. 27 An incidence of grade 1 adverse events of up to 20% of participants in both groups was considered desirable. For the interventions in the present study to be considered safe, a total absence of grades 3 to 5 adverse events was acceptable.

**Figure 1:**
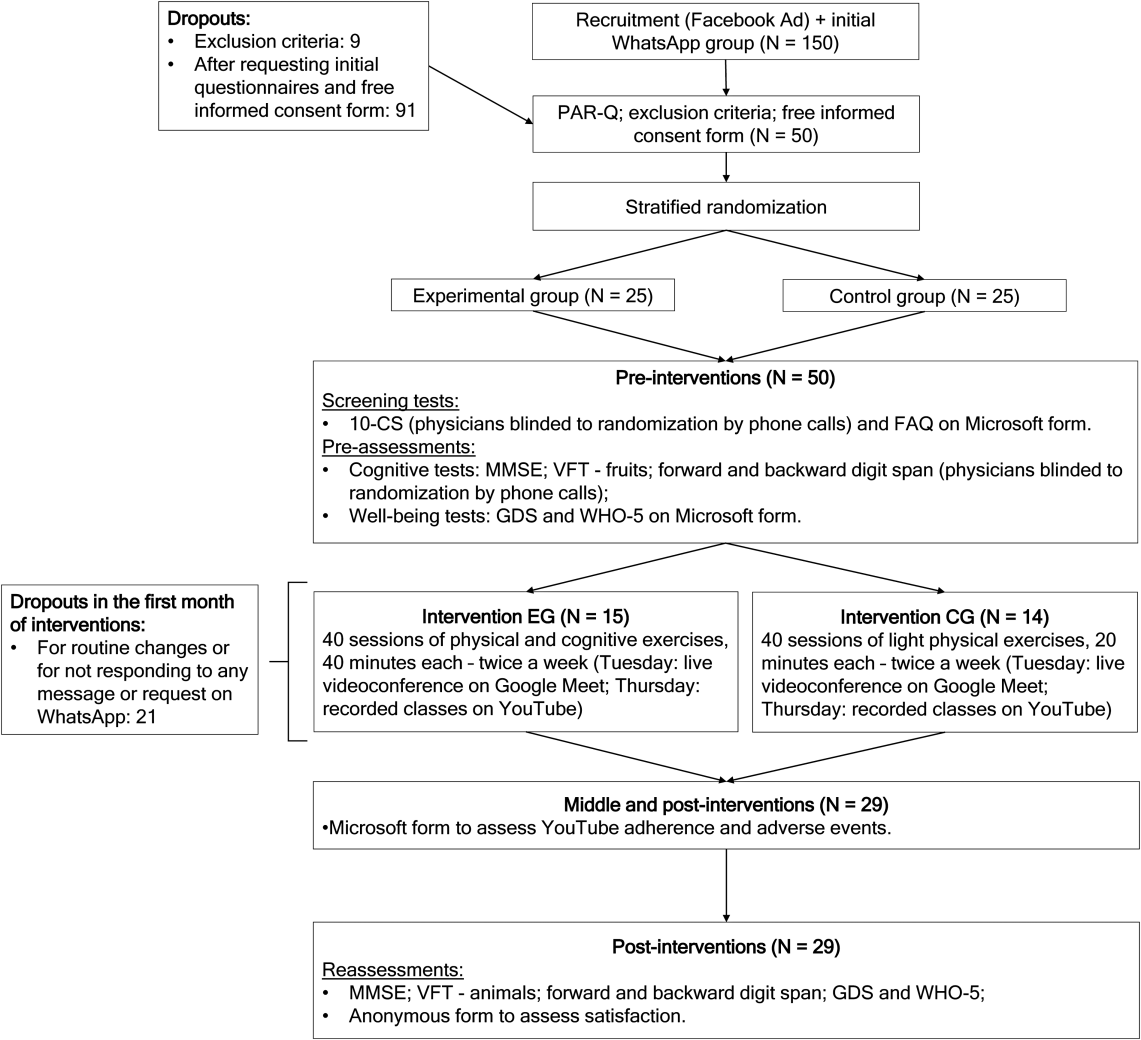
Study design flowchart.

At the end of the study, a form was sent to assess satisfaction anonymously (questions related to the exercises, researcher, session duration, difficulties, complaints, and suggestions). Answers ranged from 1 to 5: (1) terrible, (2) bad, (3) fair, (4) good, (5) great; “yes”, “no” or “sometimes” answers; or multiple choice. 16 35 The criterion for considering the program satisfactory is to obtain a minimum of 75% of answers between “good” and “excellent” in the general evaluation. The study design is illustrated in Figure 1 .

### Description of interventions

Two session formats were tested:

- Recorded classes on YouTube; and
- Live classes on Google Meet. Both groups performed 40 sessions twice a week, 20 in each format.

The EG performed 40-minute sessions of a combined protocol with cognitive, strength, aerobic, and flexibility exercises, and CG performed 20-minute light exercises sessions: stretching and joint mobilization.

The cognitive exercises were developed by the researchers and aimed to explore motor tasks that require mechanisms: information capture, cognitive functions (such as selective attention, spatial orientation, short-term memory, verbal working memory, and manipulation abilities), motor execution, and repetition. To exemplify one of the five tasks performed, the memory elements exercise consisted of memorizing an eight-word sequence and their specific ways of handling the ball.

Strength training starts with postural and muscle recruitment exercises. They performed exercises using body weight and elastic bands, increasing volume and range of motion gradually. As a home-based protocol, this was how we increased the intensity of strength exercises. Feedback was used to increase the recruitment of the muscles involved in the movement, guiding participants to induce more muscle contraction. The description of EG intervention is available in Supplementary Material ( https://www.arquivosdeneuropsiquiatria.org/wp-content/uploads/2024/02/ANP-2023.0164-Supplementary-Material.pdf ), examples of cognitive and strength exercises is available at https://www.youtube.com/playlist?list=PL4wvCDJLVmm7_Vxi8A3TCoKGNAJnDJaYy .

Aerobic training was based on rhythmic activities with multi-joint movements, with less or high impact, oriented to each participant. Exercise intensity was monitored by heart rate reserve (HRR) 36 and the rating of perceived exertion (RPE) scale. 37 The aim was to work out at 40 to 60% of HRR or 6 to 8 on a 0 to 10 RPE scale. The individual target heart rate zone was informed to participants, who monitored themselves by feeling the pulsation in the carotid artery after aerobic training.

General stretching exercises were performed, with one to two sets of 30 seconds for each position, in addition to joint mobilization exercises.

The CG intervention comprised general stretching exercises and joint mobilization in multiple articulations, each session for 20 minutes. No cognitive exercises were performed.

Both protocols are detailed in the appendix to allow for reproducibility.

### Statistical analysis

For the primary outcome, the following quantitative criteria were considered:

- Feasibility: the interventions are considered feasible if achieve the proposed goal of adherence, safety, and satisfaction. 14 15 16 17 23
- Safety: percentage of the total number of mild, moderate, and severe adverse events.
- Adherence: percentage ratio between the total number of sessions performed, in three analyses – Google Meet adherence (GMA), YouTube (YTA), and overall adherence (OA), which is the average of the other two.

A descriptive analysis of the data was presented, providing for the categorical variables the distribution of absolute ( *n* ) and relative (%) frequencies, and the main summary measures, such as measures of position and dispersion for the categorical variables. To assess the association of categorical variables in the group of participants (EG and CG), the independence test (Fisher's exact test) was applied to verify differences at baseline.

In addition, due to data distribution, the Mann-Whitney U test was used to verify differences between groups before and after the interventions for cognitive and well-being variables.

The data distribution of cognitive and well-being variables was compared between the two groups at the beginning of the study (pre-intervention) and the end (post-intervention) using the Mann-Whitney U test. The significance level was at p ≤ 0.05. SPSS software version 21 was used in all analyses.

## RESULTS

The result of the comparison of the descriptive analyses between the groups is presented in Table 1 . No significant differences between the groups were found in any of these variables.

**Table 1:** Descriptive analysis of sociodemographic, cognitive and IPAQ variables in relation to the group

Variable	Category	Group		*p* value
		CG ( *n* = 14)	EG ( *n* = 15)	
Age	Mean (SD)	66.71 (4.18)	67.8 (4.84)	0.510†
	Median (min-max)	66 (62-75)	67 (60-78)	
Level of schooling	Mean (SD)	13 (3.28)	11.73 (4.56)	0.451†
	Median (min-max)	15 (8-15)	15 (2-15)	
Level of physical activity pratice by IPAQ	Active	9 (64.3%)	10 (66.7%)	0.999*
	Inactive	3 (21.4%)	4 (26.7%)	
	Insufficiently active	2 (14.3%)	1 (6.7%)	
MMSE raw scores	Normal	14 (100%)	15 (100%)	NA
Functional activity questionnaire	Mean (SD)	0.14 (0.53)	0.60 (1.59)	0.326†
	Median (min-max)	0 (0-2)	0 (0-6)	

Abbreviations: IPAQ, International Physical Activity Questionnaire; MMSE, Mini-mental state examination; NA, not applicable; SD, standard deviation.

Notes: *Fisher's exact test; † Mann-Whitney U test.

The rate of adherence and occurrence of adverse events are shown in Table 2 . Occurrences were mild muscle pain and tiredness/fatigue after exercise. Grade 2 (moderate) adverse event occurred only in one EG participant (self-medication to prevent muscle pain). There were no severe events.

**Table 2:** Comparison between groups regarding adherence and adverse events

Variable		Group		*p* value
		CG ( *n* = 14)	EG ( *n* = 15)	
% adverse events GRADE 1		3 (21.4%)	5 (33.3%)	0.682*
% adverse events GRADE 2		0 (0%)	1 (6.7%)	0.999*
% adverse events GRADE 3-5		0%	0%	NA
% Google Meet adherence (GMA)	Mean (SD)	78.93 (15.46)	83.67 (16.42)	0.404†
	Median (min-max)	80 (55-100)	85 (45-100)	
% YouTube adherence (YTA)	Mean (SD)	69.64 (37.89)	80.83 (23.56)	0.450†
	Median (min-max)	94 (0-100)	100 (50-100)	
% overall Adherence (OA)	Mean (SD)	74.29 (19.6)	82.25 (16.11)	0.213†
	Median (min-max)	80 (35-97.5)	88 (50-100)	

Abbreviations: NA, not applicable; SD, standard deviation.

Notes: *Fisher's exact test; † Mann-Whitney U test.

Adherence was above 75% in both formats (YouTube and Google Meet) in the EG, reaching the pre-established criterion for this variable. The overall adherence was 74.29% in CG (adherence was above 75% only in the Google Meet).

Concerning satisfaction, 93% of the participants in the EG ( *n* = 14) and 93% of the CG ( *n* = 13) rated the study as “excellent,” one participant in EG and one in CG rated it as “good.”

When questioned about specific difficulties (directed questions), the answers were: 21.4% of the CG and 20% of the EG reported problems with the internet; 7.1% of the CG and 13.3% of the EG had cell phone problems; 7.1% of the CG had difficulty seeing and hearing the teacher; 7.1% of the CG and 6.9% of the EG had trouble opening the Google Meet link; 13.3% of the EG found the exercises very difficult; 6.7% of the EG found the assessments and forms too long.

### Cognitive and well-being variables

**Figure 2:**
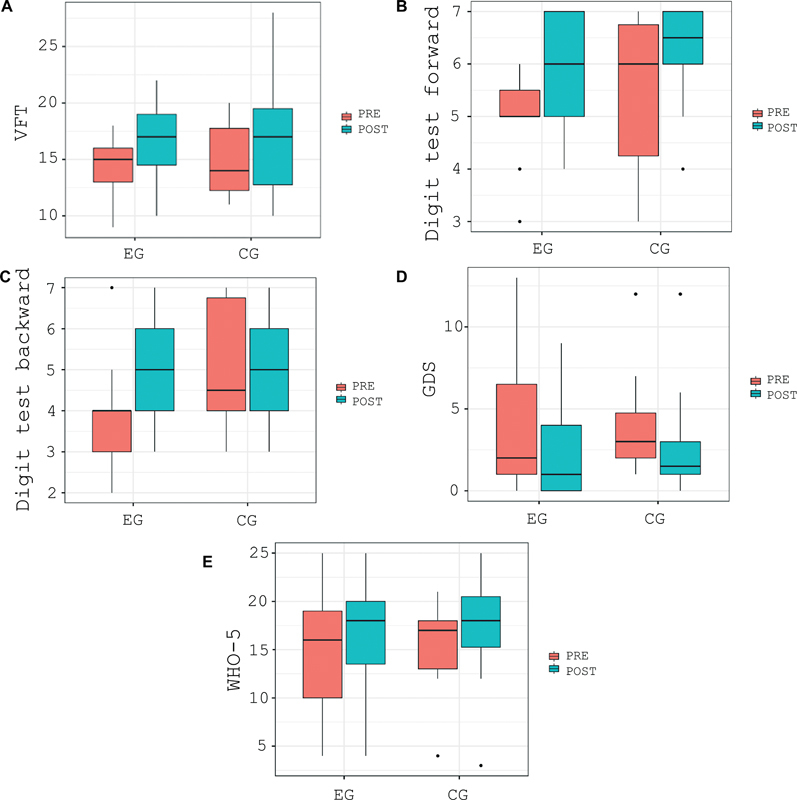
Abbreviations: VFT, verbal fluency test; EG, experimental group; CG, control group; GDS, geriatric depression scale; WHO-5, Well-being index. Box-plot of cognitive testing. A) VFT; B) Digit test forward; C) Digit test backward; D) GDS; E) WHO-5.

There was no difference between the CG and EG groups and cognitive and well-being variables when comparing the two groups at baseline and post-intervention ( Table 3 ). In the comparison of variables between the pre-and post-intervention moments in each group separately, there was an influence of activities in the EG with higher scores in the verbal fluency test and the digit test in the post-intervention, as well as a reduction in the scores on the geriatric depression scale and higher scores of well-being ( Table 4 ). The CG showed improvement only in the GDS (decrease in depressive symptoms). The variables that showed improvement with a statistically significant difference in both groups are illustrated in box-plot graphs ( Figure 2 ).

**Table 3:** Cognitive and well-being variables between groups pre and post

Variable	Group	*n*	Median	Mean	Standard deviation	*p* value*
MMSE PRE	EG	15	20.00	19.93	1.22	0.404
	CG	14	21.00	20.21	1.48	
MMSE POST	EG	15	20.00	20.00	1.51	0.165
	CG	14	21.00	20.57	1.79	
VFT PRE	EG	15	15.00	14.27	2.37	0.709
	CG	14	14.00	14.86	2.96	
VFT POST	EG	15	17.00	16.60	3.20	0.965
	CG	14	17.00	17.14	5.46	
Digit test forward PRE	EG	15	5.00	5.00	0.85	0.214
	CG	14	6.00	5.50	1.34	
Digit test forward POST	EG	15	6.00	5.80	1.21	0.367
	CG	14	6.50	6.21	0.97	
Digit test backward PRE	EG	15	4.00	3.93	1.49	0.095
	CG	14	4.50	4.93	1.59	
Digit test backward POST	EG	15	5.00	5.13	1.36	0.751
	CG	14	5.00	5.00	1.57	
GDS PRE	EG	15	2.00	4.20	4.23	0.741
	CG	14	3.00	3.71	2.92	
GDS POST	EG	15	1.00	2.67	3.24	0.624
	CG	14	1.50	2.71	3.15	
WHO-5 PRE	EG	15	16.00	14.60	6.82	0.63
	CG	14	17.00	15.71	4.46	
WHO-5 POST	EG	15	18.00	16.60	6.05	0.81
	CG	14	18.00	17.21	5.47	

Abbreviations: GDS, Geriatric depression scale; MMSE, Mini-mental state examination; VFT, verbal fluency test; WHO-5, well-being index.

Note: *Mann-Whitney U Test.

**Table 4:** Comparison between pre and post-intervention in the CG and EG groups

	MMSE		VFT		Digit test forward		Digit test backward		GDS		WHO-5	
	PRE	POST	PRE	POST	PRE	POST	PRE	POST	PRE	POST	PRE	POST
EG (median)	20	20	15	17	5	6	4	5	2	1	16	18
*p* value	0.66		0.025		0.028		0.011		0.021		0.032	
CG (median)	21	21	14	17	6	6	4	5	3	2	17	18
*p* value	0.546		0.257		0.083		0.807		0.005		0.081	

Abbreviations: CG, control group; EG, experimental group; GDS, EGriatric depression scale; MMSE, Mini-Mental State Examination; VFT, verbal fluency test; WHO-5, well-being index.

Note: *Wilcoxon test.

## DISCUSSION

The present study reached the pre-established criteria to demonstrate its feasibility and confirmed the hypothesis. The groups completed the entire period of interventions with high adherence and excellent satisfaction levels, in addition to presenting few adverse events, without compromising safety criteria.

### Safety

The incidence of grade 1 adverse events (mild symptoms) was very close to the desirable (up to 20%) in the CG (21.4%) and above the desirable in the EG (33.3%). One person from the CG and five from the EG reported at least one episode of mild muscle pain and/or tiredness/fatigue after the exercise session, with no restrictions on function or need for intervention. Among the seven people who presented such symptoms, three from the EG and two from the CG were classified as physically inactive, and two from the EG and one from the CG were active, according to the IPAQ. The occurrence of these grade 1 adverse events does not pose a threat to the safety of the study. Safety in the study is determined by the absence of serious adverse events. 13 35 38

The incidence of grade 2 (moderate) adverse events occurred in one person previously inactive from the EG, who reported taking analgesics after the session. The participant justified the use of the drug as a preventive way to avoid pain. The incidence of grade 2 adverse events was within the previously established limit. There were no 3 to 5-grade adverse events.

### Adherence

Adherence is considered positive in obtaining a minimum average of 75% participation in the sessions offered (Google Meet and YouTube). An important aspect of being considered in adherence is the high number of dropouts in the sample size. 64.53% of eligible people dropped out before the randomization process without informing the reasons. Regarding the sample size of the feasibility studies with remote protocols, such losses are reported, and the number of older adults included in the analyses is small. 14 15 16 17 23

Regarding adherence to the intervention sessions, only the EG group reached the established criterion, above 75% in both session formats (YouTube and Google Meet). The average overall adherence was 74.29% for the GC and 82.25% for the EG. Although the EG had a higher mean of adherence than the GC, there was no significant difference between the groups, and it is not possible to say that the type of intervention influenced the result of adherence.

Many previous feasibility studies documented remote strategies. 14 15 16 17 23 39 40 The adherence found refers to interventions that allowed flexibility in the execution times, which is an advantage for routine adjustment and adaptation for occasional appointments without real-time interaction. Half of the overall adherence found in both groups was dependent on schedule; in addition to the absence of connection problems (Google Meet sessions), the result is satisfactory.

### Feasibility

Access to technology is the first criterion to enable the feasibility of intervention processes, regardless of their format. Inviting the participants to the study constitutes a natural exclusion since the opportunity comes only to people who have a smartphone or mobile data plan, are on the social network Facebook, and, possibly, on the WhatsApp application. In the study, no participant was guided or helped to install the WhatsApp application or Google Meet.

In addition to access to technology, safety, and adherence, an important aspect to consider in this study of the feasibility of the remote exercise protocol is satisfaction with the intervention. When considering the overall evaluation of the program, 100% of the participants in both groups rated it between good and excellent, which indicates a positive result.

A significant advantage of remote interventions is the possibility of reaching people in other territories, which allows social bonds between people with different customs and realities, in addition to the benefits of the intervention itself. Almost half of the participants were from São Paulo; the others were from sixteen other cities in nine different Brazilian states, which allows us to say that the scope was national and can be expanded.

### Cognitive and well-being variables

The comparison between the pre-and post-intervention moments in cognitive variables did not present a significant difference between the groups. When analyzing each group, only the EG showed a significant improvement in verbal fluency and digit tests. The cognitive exercises performed by the EG may have contributed to the results obtained in this group.

In the well-being variable obtained by the WHO-5 instrument, the EG group showed improvement and acquired lower depression scores. The CG only had an improvement in the scale that assesses the presence of depressive symptoms. The political, economic, and social aspects that Brazil is still facing during the period in which this study was carried out may contribute to the increase in “yes” answers in this instrument at the baseline. According to the scale, 30 the cutoff score for the presence of depressive symptoms is 5, and in the baseline, the average for the EG was 4.2, and for the CG, 3.7.

In conclusion, the study contemplated the pre-established criteria for the safety and adherence of the remote exercise program offered, as well as reached the desired degree of satisfaction with reports of difficulties that were not representative and did not prevent access to interventions, the feasibility of this remote protocol of physical exercises and cognitive skills proved to be sustainable.

The results suggest that the combined protocol between physical and cognitive exercises (EG) has a higher potential to impact cognitive functions such as verbal fluency and attention positively, and both interventions were beneficial in improving the subjective perception of well-being.

This study has proved the feasibility of an online intervention in a low- and -middle-income country, with an important role in public health.

## Limitations

Since this study only included women with a high level of education, it was not possible to infer the results for the population of both genders with low education. Due to a very small sample size and the lack of a control group, our results should be considered as exploratory. As we focused on feasibility, security, and adherence, the physical impact of the exercises was not assessed. Future studies could investigate the effects on physical function we can expect from remote exercise protocols, using ANOVA to analyze the presence of a possible interaction between intervention time and group.
