# Supplementary material for: Feasibility, safety, and adherence of a remote physical and cognitive exercise protocol for older women
Source: Arq Neuropsiquiatr. 2024 Apr 23;82(4):s00441785690. doi: 10.1055/s-0044-1785690 (PMC11039073; doi:10.1055/s-0044-1785690)
Supplement: Supplementary file 1 — Supplementary Material [file 10-1055-s-0044-1785690-s230164.pdf]

Appendix - EG – Schedule of sessions

EG Sessions 1 - 40

	1	2	3	4	5	6	7	8	9	10
**Part 1**	Introduction 1 Safety / educational exercises	Introduction 2 Safety / educational exercises	Introduction 3 Posture / join alignment	Introduction 4 Posture / join alignment	Teaching Abs contraction	Revision Abs contraction	Teaching Mesure heart rate	Revision Mesure heart rate	Teaching Use of the Digital ID	Teaching Hands 1 2 3
**Part 2**	ME 1 - MI 1 Session 1/4	ME 1 - MI 1 Session 2/4	ME 1 - MI 1 Session 3/4	ME 1 - MI 1 Session 4/4	ME 1 - MI 2 Session 1/4	ME 1 - MI 2 Session 2/4	ME 1 - MI 2 Session 3/4	ME 1 - MI 2 Session 4/4	ME 1 - MI 3 Session 1/4	ME 1 - MI 3 Session 2/4
	11	12	13	14	15	16	17	18	19	20
**Part 1**	Revision 1 Hands 1 2 3	Teaching Peloton pace	Revision 1 Peloton pace	Teaching Memory elements	Revision 1 Memory elements	Revision 2 Hands 1 2 3	Revision 2 Peloton pace	Revision 2 Memory elements	Teaching Dial extension	Revision 1 Dial extension
**Part 2**	ME 1 - MI 3 Session 3/4	ME 1 - MI 3 Session 4/4	ME 1 - MI 4 Session 1/4	ME 1 - MI 4 Session 2/4	ME 1 - MI 4 Session 3/4	ME 1 - MI 4 Session 4/4	ME 2 - MI 1 Session 1/4	ME 2 - MI 1 Session 2/4	ME 2 - MI 1 Session 3/4	ME 2 - MI 1 Session 4/4
	21	22	23	24	25	26	27	28	29	30
**Part 1**	Teaching Three balls	Revision 1 Three balls	Teaching Leg coordination	Revision 1 Leg coordination	Revision 2 Dial extension	Revision 2 Three balls	Revision 2 Leg coordination	Practice Hands 1 2 3	Practice Hands 1 2 3	Practice Peloton pace
**Part 2**	ME 2 - MI 2 Session 1/4	ME 2 - MI 2 Session 2/4	ME 2 - MI 2 Session 3/4	ME 2 - MI 2 Session 4/4	ME 2 - MI 3 Session 1/4	ME 2 - MI 3 Session 2/4	ME 2 - MI 3 Session 3/4	ME 2 - MI 3 Session 4/4	ME 2 - MI 4 Session 1/4	ME 2 - MI 4 Session 2/4
	31	32	33	34	35	36	37	38	39	40
**Part 1**	Practice Peloton pace	Practice Memory elements	Practice Memory elements	Practice Dial extension	Practice Dial extension	Practice Three balls	Practice Three balls	Practice Leg coordination	Practice Leg coordination	Final celebration
**Part 2**	ME 2 - MI 4 Session 3/4	ME 2 - MI 4 Session 4/4	ME 3 - MI 1 Session 1/4	ME 3 - MI 1 Session 2/4	ME 3 - MI 1 Session 3/4	ME 3 - MI 1 Session 4/4	ME 3 - MI 2 Session 1/4	ME 3 - MI 2 Session 2/4	ME 3 - MI 2 Session 3/4	ME 3 - MI 2 Session 4/4

ME = Mesocycle (exercises program);
MI = Microcycle (exercises volume - sets and reps)

Part 1 – cognitive exercises description

- **Use of the digital ID**

As a safety and monitoring strategy, each participant in this group received their digital ID (identifier), an image containing their name, the target heart rate zone measured in 15 seconds, and codes with shapes and colors that represent pathologies that may cause adverse events due to the practice of exercises. Participants do not know what exactly they mean, but they were taught to adapt or heed a certain orientation on an aspect of movement or posture, if they had a specific code.

To exemplify this process, during the "lunge", which is an exercise for the lower limbs that is performed with anteroposterior separation of the legs, the researcher tells the "people who have the green color in the ID" to perform the movement with less amplitude. The green color represents pain in the knees, and reducing the range of motion is necessary to reduce patellofemoral compression from the exercise. For this reason, the images are in the possession of the participants during the sessions, as they are the ones who listen to the general orientation and attend to it individually, if necessary. The IDs were formulated after filling in the health data present in the monitoring questionnaire.

- **Hands 1 2 3**

The researcher teaches and asks for the memorization of the sequence of commands 1, 2 and 3, as follows:

Command 1 – Clap your hands;

Command 2 – Snap your fingers;

Command 3 – Show the palms of your hands.

Then, the researcher speaks the numbers, first sequentially, then randomly, without repeating the movements.

In a third phase, the researcher makes a "virtual duo" with the participants, starting by speaking and doing command 1, and each participant must give the sequence, speaking and doing command 2, and the turn returns to the researcher, who will do command 3, and so on.

- **Peloton pace**

The participants were instructed to start and maintain a stationary gait, involving movement of the lower and upper limbs. While the gait was maintained, they repeated the commands given in a rhythmic manner by the researcher in the reverse order, without interrupting the gait. For example, the teacher dictated "1, 2, 3, 4", and the participants started again at the end at "4, 3, 2, 1". Four different combinations were used: "1, 2, 3, 4"; "4, 3, 2, 1"; "1, 3, 2, 4" and "1, 4, 3, 2".

Throughout the activity, the participants remained with the microphones closed and the researcher gave the time that would be enough to recall the reverse order, even considering a slower pace. After this time, the

researcher dictated the reverse order and instructed the participants to make a self-assessment of performance (hit or miss).

In the last stage of the activity, the researcher approached the camera and nominally divided them into four groups. Each group, separately, evoked the reverse order to the one said by the researcher using the fingers of the hands. Thus, the researcher had a visual reference of hits and misses for individual guidance.

- **Memory elements**

This task requires participants to memorize two semantic categories, colors, and animals, with their sequence of words and associating them with the specific movement of each one. The material used is a medium rubber ball, which if the participant does not have it at home and cannot borrow from a child, she can use a ball made with socks. All the movements performed on each word end with a throw and receive of the ball, either to a wall or upwards.

**1. colors**	**1.**	**Blue:** throws the ball upwards
	**2.**	**Yellow:** throws the ball up
	**3.**	**Green:** throws the ball with the right hand
	**4.**	**Red:** throws the ball with the left hand
**2. animals**	**5.** 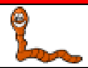	**Worm:** bounce the ball
	**6.** 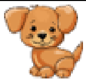	**Dog:** put the ball on your knee
	**7.** 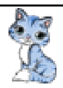	**Cat:** flex one knee and throws the ball upwards
	**8.** 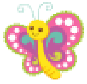	**Butterfly**: ball behind the back and throws upwards

- **Dial extension**

The task begins with the construction of a spatial scheme: the participants were instructed to position themselves with their feet together at a starting point. They were then instructed to visualize a digital telephone keypad on the floor, with nine keys, three in the front row, three in the middle row, and three in the back. The researcher demonstrated, from left to right (as it reads), the position of each digit: in the front row, digits 1, 2 and 3; in the middle 4, 5 and 6; in the back 7, 8 and 9. Therefore, they started positioned in the center, on the number five key.

After visualizing these positions, the researcher divided the participants into four groups, dictated a 3-digit extension and asked each group to perform separately by stepping with both feet on each key. The movement of the participants, starting from the same key, should be the same on the screen in case of a correct answer. It was possible for the researcher to visualize errors in the execution when the participants did not move or directed themselves to the wrong side. A total of three extensions were given, one at a time. At the end of the activity, the researcher approached the camera and asked them to show each of the requested extensions with their fingers.

- **Three balls**

This activity used a medium rubber ball, or a ball made with socks. The researcher taught three ways to combine ball manipulation with leg movement. In command 1, the movement was to throw the ball from one hand to the other, forming a parabola in the air. In command 2, bounce the ball on the ground, receiving the ball with the other hand. People who didn't have the ball at home and used a sock ball, executed command 2 by throwing the ball up. In command 3, the move was to throw the ball upwards with the body on one side and switch sides to receive it. Then, a palm was added before the ball returned to the hand, in an interspersed manner: sometimes in command one, sometimes in two, sometimes in three.

- **Leg coordination**

This task involved visualizing a square on the floor, and the participants started with both feet inside the square. Next, the researcher taught the rule of leg movement: the right leg always moves backwards, and the left always forwards. The body weight should remain within the square, as a base of support, and the legs move alternately, sometimes right, sometimes left, in their respective directions, only touching the tip of the foot outside the square.

The next phase consisted of "mirroring" the movement, that is, the researcher started with one leg and the participants reacted by moving the opposite leg, fitting the movement. For example, if the researcher moved the right backwards, this forces the participants to move the left forward. After the first movement, the others alternated right and left, in the same rhythm, until the researcher stopped and started again, choosing her starting leg again. In this phase, the movements were paused to allow the correction of laterality, when necessary.

Part 2 – strenght exercises description

- **Mesoclycle 1**
  1. Squat
  2. Standing hip flexion
  3. Standing hip abduction
  4. Standing calf raises
  5. Shoulder abduction

- **Mesoclycle 2**
  1. Squat
  2. Standing leg extension
  3. Vertical row (rubber band)
  4. Chest press (rubber band)
  5. External rotation (rubber band)
  6. Lower Abs on chair

- **Mesoclycle 3**
  1. Lunges
  2. Hip flexion + hip abduction
  3. Standing leg curl
  4. Hip thrusts on chair
  5. Press ups
  6. Vertical row (rubber band)
  7. 30 second's plank

Progression of microclycles

Microcycle 1	2 sets of 10 reps
Microcycle 2	2 sets of 12 reps
Microcycle 3	3 sets of 10 reps
Microcycle 4	3 sets of 12 reps

Lesson plan examples (Each session has a complete lesson plan. Contact the authors to access the others)

Introduction 1 and 2

**Part 1 – Cognitive exercises (10')**	**Goal:** safety / educational exercises
	**Strategy:** INTRODUCTION – BRAIN, MUSCLE CONTRACTION – session 1/2
	**Material:** Without Material
	**Description:**<br><br>SAFETY: Talk about safety – breakfast, bottle of water as part of our class material! Symptoms that I could expect to feel and others I must report to the researcher.<br><br>Explain about the purpose of part 1 of the session = Cognitive. Discuss the brain, the decrease of many functions and the need to stimulate these capacities. For this, all exercises must be done with maximum attention, focus, awareness. We must remember that all the movements of the body are commanded by the brain! The muscles only move by brain command.<br><br>Let's give neural command to our muscles now! I'm going to say a body part, a muscle (name it and show where it is), and we're going to contract that musculature! The sensation of contracting is of stiffening, the muscle "jumps", gets hard, warms up. Explore the muscles from the bottom up, from the feet to the hips. The emphasis is on muscle contraction of the soles of the feet, gastrocnemius, tibialis anterior, quadriceps and glutes.
**Part 2: Strength exercises (10')**	**Strategy:** Resistance exercises
	**Material:** Body Weight
	**Exercises:** teach joint alignment, contraction movement, and breathing in each of the movements below. Point out where it is to feel and what to feel. Explain the difference in pain and muscle effort, teaching how to adjust the amplitude by the pain threshold.<br><br>2 sets of 10 reps each<br><br>1. Squat<br>2. Standing hip flexion<br>3. Standing hip abduction<br>4. Standing calf raises<br>5. Shoulder abduction
**Part 3: Aerobic exercises (10')**	**Strategy:** Rhythmic Activity
	**Material:** Music. Suggestion - Staying alive; New York New York...
	**Exercise:**<br><br>**Phase 1:** Walking through space, ask participants to pay attention to the correct walking technique: touch the ground with your heel, pass your body weight along the entire axis of your foot and lift it off the ground by your fingertips. Never enter with the front of the foot on the ground. Let people walk for a while giving these directions and different ways to walk and to touch the ground.<br><br>**Phase 2**: Teach joint movements as they walk: flexion, extension, and circumduction of the ankles; flexion and extension of the knees; flexion, extension, abduction, adduction, and circumduction of the hips; flexion and rotation of the spine (gently). Keep observing, perceiving the muscle responses.<br><br>At the end, stop where you are, bring your hands to your heart, notice the sensations of the body. If you have increased your heart rate, breathing, temperature, if there was pain, if there was any discomfort and so on.
**Part 4: Flexibility (10')**	**Material:** Music selection – soft songs
	**Exercises:** Teach correct postural alignment, point out the muscles that should be stretched gently.<br><br>Perform one to two sets of 30 seconds each, for the main muscles mentioned below: Gastrocnemius, Iliopsoas, Hamstrings, Quadriceps, Pectorals, Latissimus dorsi, Deltoids.<br><br>You can insert complementary stretching, joint mobilization and breathing exercises.

Introduction 3 and 4

**Part 1 – Cognitive exercises (10')**	**Goal:** safety / educational exercises
	**Strategy:** INTRODUCTION – Posture / join alignment – session 1/2
	**Material:** Without Material
	**Description:**<br><br>Start the postural organization from head-to-toe distance of the feet a little greater than the hip, parallel feet, pay attention to the distribution of the weight of the body on the soles of the feet, without unequal weight falling between heels and toes. Bring attention to the knees, keeping them relaxed, without exaggerating the flexion or stiffening. Show the difference between x-knee (valgus) and knight's knee (varus) and show that the knees need to be aligned with the ankles in postures and exercises. Remember to tone your muscles in the meantime. Moving up to the movements of the pelvis: anteversion, retroversion. Seek neutral alignment of the lumbar spine.
**Part 2: Strength exercises (10')**	**Strategy:** Resistance exercises
	**Material:** Body Weight
	**Exercises:** teach joint alignment, contraction movement, and breathing in each of the movements below. Point out where it is to feel and what to feel. Explain the difference in pain and muscle effort, teaching how to adjust the amplitude by the pain threshold.<br><br>2 sets of 10 reps each<br><br>1. Squat<br>2. Standing hip flexion<br>3. Standing hip abduction<br>4. Standing calf raises<br>5. Shoulder abduction
**Part 3: Aerobic exercises (10')**	**Strategy:** Rhythmic Activity
	**Material:** Music selection – upbeat songs
	**Exercise:**<br><br>Joint movements (gait, hip flexion, hip abduction, knee, and elbow flexion).<br><br>**Drink water between sets and ask them to drink it too.**
**Part 4: Flexibility (10')**	**Material:** Music selection – soft songs
	**Exercises:** Teach correct postural alignment, point out the muscles that should be stretched gently.<br><br>Perform one to two sets of 30 seconds each, for the main muscles mentioned below: Gastrocnemius, Iliopsoas, Hamstrings, Quadriceps, Pectorals, Latissimus dorsi, Deltoids.<br><br>You can insert complementary stretching, joint mobilization and breathing exercises.
